# Beckwith-Wiedemann Syndrome: Open bite evolution after tongue reduction

**DOI:** 10.4317/medoral.21319

**Published:** 2018-02-25

**Authors:** Estefanía Alonso-Rodriguez, Elena Gómez, Mercedes Martín, Jesús-Manuel Muñoz, Juan Hernández-Godoy, Miguel Burgueño

**Affiliations:** 1 M.D Department of Oral and Maxillofacial Surgery, Hospital Universitario La Paz, Madrid, Spain; 2 M.D Department of Plastic and Reconstructive Surgery and Burn Unit, Hospital Universitario La Paz, Madrid, Spain; 3 M.D., Ph.D. Department of Oral and Maxillofacial Surgery, Hospital Universitario La Paz, Madrid, Spain. Chief of Department

## Abstract

**Background:**

Macroglossia causes functional deficits such as airway obstruction, drooling, phonation difficulties, and leads to protrusion of dentoalveolar structures resulting in an anterior open bite and a prognathic mandibular appearance. Macroglossia is present in the majority of patients with Beckwith-Wiedemann syndrome (BWS) and surgical treatment may be indicated.

**Material and Methods:**

A retrospective review was conducted including BWS patients who underwent surgical tongue reduction between 2000 and 2015 at the Hospital Universitario La Paz, Madrid.

**Results:**

Out of 16 patients with BWS, surgery was performed in 11 cases. Tongue protrusion with open bite was the main indication for surgical treatment. Reduction glossectomy was performed using the keyhole technique. We analysed the relationship between age at surgery and evolution of open bite. Complications were minimal and satisfactory outcomes were observed with a decrease in anterior open bite.

**Conclusions:**

In this study we have observed that surgical treatment in patients with BWS and open bite accompanied by macroglossia seems to provide positive results with a satisfactory outcome in dentoskeletal alterations.

** Key words:**Macroglossia, Beckwith- Wiedemann syndrome, glossectomy; tongue reduction, malocclusion.

## Introduction

Beckwith-Wiedemann Syndrome (BWS) is a congenital overgrowth disorder with an estimated incidence of approximately 1 in 13,700 to 17,000 births (1,2). This rare syndrome was first described by Beckwith in 1963 and Wiedemann in 1964. It originates from chromosomal changes in the imprinted 11p15.5 region, where multiple genes related to growth produce increased levels of the fetal insulin-like growth factor 2 (IGF-2). Out of the total number of cases, 85 % are sporadic and 15% are familial (3,4).

The typical triad consists of exomphalos, macroglossia and gigantism. They have an increased risk of developing embryonic malignancies. However, clinical features are variable and many diagnostic findings and scoring systems have been described (5–7). Head and neck manifestations seen in BWS include facial naevus flammeus, anterior earlobe creases and posterior helical pits, prominent occiput, cleft palate, microcephaly or flattened nasal dorsum. Macroglossia, however, remains the most consistent symptom and is seen in 80-99% of patients (3,8). Macroglossia is a phenotypically heterogeneous condition with different degrees of severity and a surgical intervention will not always be necessary. Enlargement of the tongue results from hyperplasia of muscle fibers and is generally increased in all three dimensions (9,10).

The resting pressure of the tongue against the teeth may lead to the development of malocclusion, resulting in a dental class III, an anterior open bite, prognathism and interdental spaces. Macroglossia may give the appearance of mental retardation, even though these patients very often have normal mentation and intellectual delay is seen in only a minority of cases, most of which are related to perinatal hypoglycemias.

Our objective is to analyse the anterior open bite evolution in these patients after surgery.

## Material and Methods

We report a retrospective review of all patients diagnosed with BWS between 2000 and 2015 at the Hospital Universitario La Paz in Madrid, Spain. Indications for tongue surgery, age at time of surgery, operation time, complications, length of follow-up, outcomes after surgery and evolution of orofacial alterations were evaluated.

## Results

Over the 15 years, 16 patients were diagnosed with BWS. Currently, 2 of these patients are awaiting intervention; 3 cases of mild macroglossia did not require surgery and 11 patients underwent tongue reduction for various different conditions, all of which had an open bite at the time of surgery. In these 11 patients, 20 partial glossectomies were performed, with an average of 1.8 surgical interventions per patient (range 1-4). The main indications for tongue surgery were open bite in 65% of patients (13 cases), airway obstruction in 15% (3 cases), oral incompetence with persistent drooling in 10% (2 cases), feeding problems in 5% (1 case) and phonation difficulties in 5% (1 case) ( Table 1).

**Table 1:**
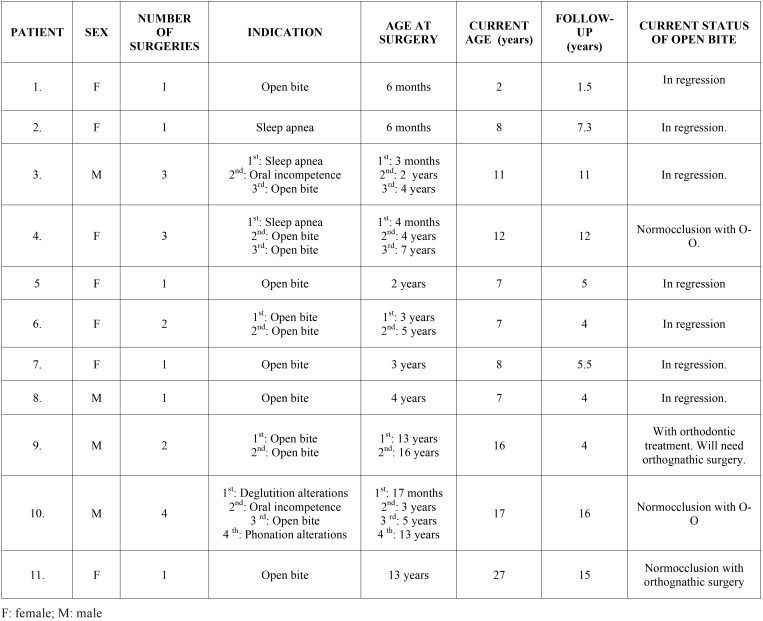
Patient characteristics.

**Figure 1:**
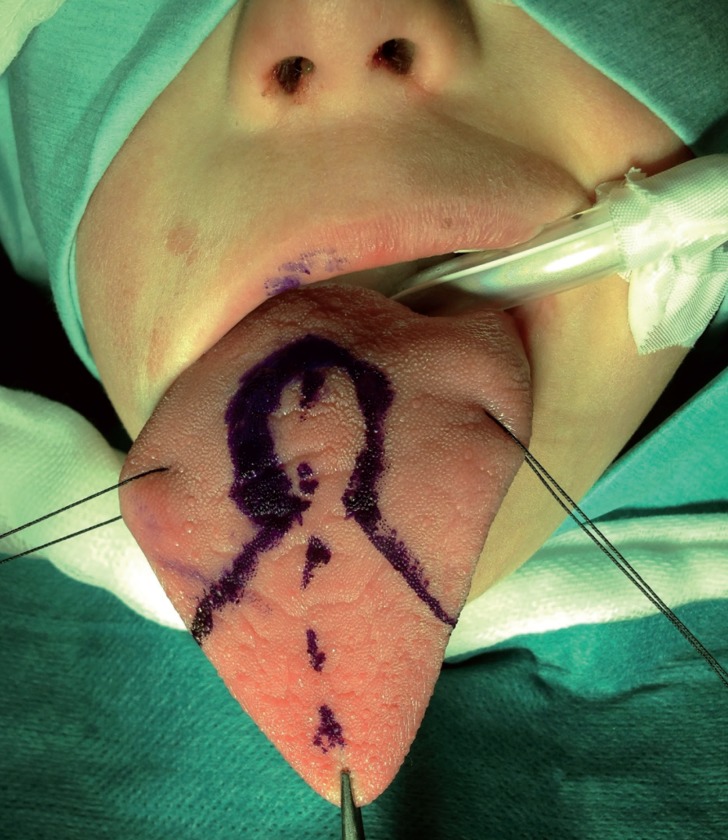
Intraoperative image. Surgical tongue reduction design.

In the majority of reported cases the keyhole technique was performed for tongue reduction. This technique allows a uniform reduction, maintains normal tongue shape and preserves the inferolateral neurovascular bundles. This technique is a combined technique, with resection of a midline portion and an anterior wedge resection (Fig. 1).

A single wound dehiscence was the only complication we observed. This case was resolved with resuturing of the dehiscence. Steroids were administered to all patients and they underwent prolonged intubation postoperatively to protect the airway from postoperative oedema.

**Figure 2:**
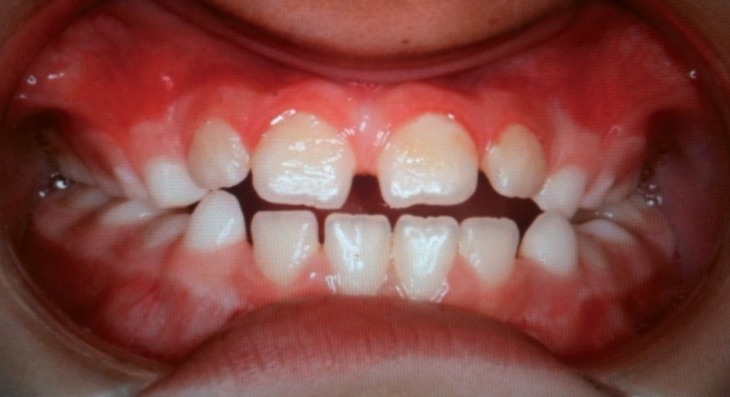
One patient from group B. This patient has been operated on 3 times. Open bite and interdental spaces before starting orthodontic treatment.

**Figure 3:**
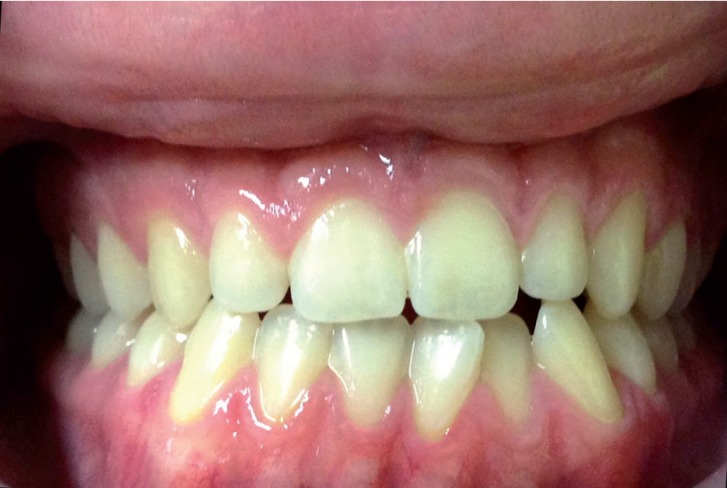
One patient from group B. This patient has been operated on 4 times. He has achieved normocclusion after orthopaedic- orthodontic treatment.

The mean follow-up period was 7.8 years (range 1.5-16 years). The mean age at the time of the first surgery, regardless of the indication, was 3.7 years (range 3 months- 13 years). Similarly, the mean age at surgery as indicated by malocclusion was 5.1 years (range 6 months-13 years). Currently, 7 of the 11 patients that underwent surgery show clinical signs of open bite regression (group A). These patients in particular are all under the age of 11 and although they have not yet started orthodontic treatment, their open bite is gradually decreasing following tongue reduction. It is difficult to objectively measure the reduction in open bite because many patients have been operated on numerous times. Furthermore, some of them underwent surgery at a very early age and the facial changes that accompany physical growth will likely have had an impact. Despite the challenge of quantifying the reduction with precision, we have observed a regression in open bite clinically. Out of the remaining patients, all of whom are over eleven, 2 of them have reached normocclusion after orthopaedic- orthodontic treatment (group B) (Figs. 2,3), and the other 2 needed (1 patient) or will need (1 patient) orthodontic treatment plus orthognathic surgery to achieve a normocclusion (group C). Mean age at the time of the first surgery in group A, with open bite in regression, was 1.9 years. In comparison, for those over the age of 11 with normocclusion after orthopaedic-orthodontic treatment (group B) this was 10.5 months. Finally, in the group requiring orthognathic surgery (group C) the mean age was 13 years, with advanced facial growth and established facial deformities at the time of tongue reduction ( Table 1).

## Discussion

Macroglossia is defined as a resting tongue that protrudes beyond the teeth or alveolar ridge. The diagnosis of macroglossia is based on subjective criteria when there is a discrepancy between the size of the tongue and the size of the oral cavity. There are no objective techniques used in the clinical diagnosis and a tongue reduction will not always be required. The main indications for surgery are: airway obstruction or sleep apnea, in such cases it is important to exclude tonsillar or adenoidal hypertrophy as the cause of obstruction; feeding problems, during the oral preparatory phase there is persistent tongue protrusion and difficulty manipulating the bolus, problems which are usually eliminated with the surgery (11); recurrent lingual trauma; persistent drooling that causes skin irritation with perioral infections and bad odour; phonation alterations on articulation, speech intelligibility and oral behaviour (12); aesthetic and psychological impact due to a false appearance of mental retardation; and dental deformities with an anterior open bite, mandibular prognathism and class III malocclusion.

Many different designs of surgical tongue reduction have been advocated. The main objective is to provide an adequately aesthetic and functional tongue whilst preserving the neurovascular bundles. These techniques can be divided into the following: peripheral excisions, which often result in a globular and immobile tongue overall if there is a tip amputation; anterior wedge resection, that decreases the length but not the width; central reductions that decrease the width but not the length; dorsal flap excisions; and combinations such as keyhole reductions, where a midline portion and an anterior wedge reduction are combined. This technique was first described by Morgan *et al.* (13) and some modifications have been described posteriorly. Keyhole reduction resembles the name of the procedure and it allows a decrease in the width, length and height. If excessive tissue remains, the mid portion of both lateral flaps may be cored out (“pita bread manoeuvre”) but this can interfere with postoperative mobility (14). None of our cases required any tissue to be cored out in order to achieve an appropriate tongue reduction. Patients with BWS, although there are individual variations, usually have a tongue enlarged in three dimensions and for this reason we have employed this technique in most of our cases.

The origin of dentofacial deformities in this syndrome is unclear. Many authors advocate that malocclusion, open bite and prognathism are only secondary to the effects of macroglossia and are not attributable to somatic gigantism or growth hormone effects (15). Kawafuji *et al.* (16) analyse seven patients with BWS that had not undergone surgery and they report that an enlarged mandibular body might also be due to mandibular cartilaginous growth activated by IGF-2 expression. However, whatever the exact origin of these dentofacial deformities, macroglossia and malocclusion are related, and the treatment of macroglossia is necessary to treat malocclusion. Several articles even describe the spontaneous correction of an anterior open bite after tongue reduction (17,18). Malocclusion is not an absolute indication for tongue reduction, but is frequently a relative indication. In our case series, this indication has been the most frequent.

The best age at which to perform surgery in order to prevent dentofacial deformities is a controverted subject. Kawafuji *et al.* (16) demonstrated that untreated macroglossia may result in an anterior open bite and a wide dental arch. Kadouch *et al.* (19) operated on patients over the age of 3 months with severe macroglossia to reduce orofacial growth alterations. However, some authors advocate that there is a risk of significant tongue regrowth after surgery when a patient is operated on too early and it can lead to reoperations. Tomlinson *et al.* (20) have reported that this may be due to relatively high levels of IGF-2 during the neonatal period and surgery should be delayed beyond the age of 6 months in the majority of cases where possible.

In our case series we have observed that in patients operated on later (group C), when orofacial deformities were well established at the age of 13, the open bite was not modified following tongue reduction and that they needed or will need not only orthodontic treatment to achieve normocclusion, but also orthognathic surgery. In these cases, tongue reduction was carried out to prevent recurrence after orthognathic surgery. The other patients, all with open bites before surgery (groups A and B), were operated on much earlier. A few patients within these groups presented with an open bite associated to sleep apnea. Unlike group C, the two other groups modified their open bite after tongue reductions. In group A, we have observed clinical improvements with regards to open bite regression. Similarly, patients in group B have achieved normocclusion with orthodontic treatment without the need for orthognathic surgery. In keeping with what has been stated above, patients operated on early do have more reoperations. Based on our experience, the age at which tongue reduction is performed depends on the indications but, in order to prevent orofacial deformities, early intervention must be considered, taking into account the possibility of recurrence and the risks of general anaesthesia.

There are few studies about BWS and evolution after tongue reduction reported in the literature (19-22). Considering that BWS is a very rare syndrome, in this study we have presented a large case series. Some of the limitations of this study are its retrospective nature and the small number of subjects, which do not allow us to draw detailed conclusions, the difficulty had in attributing an objective reduction in open bite (mm) to the treatment itself, due to age at the time of surgery, the multiple interventions required and the changes brought about by growth which may have influenced outcomes and, finally, the large range of follow-up. Further studies are needed in this area and long-term outcomes need to be evaluated.

## Conclusions

Macroglossia is a common manifestation in patients with BWS and in many cases surgery will be indicated. Macroglossia is related to malocclusion and, if untreated, an anterior open bite and dentofacial alterations may appear. To prevent orofacial deformities, an early intervention could be considered. Surgical treatment in patients with BWS and open bite with macroglossia seems to produce positive results with satisfactory outcomes in dentoskeletal alterations.
